# Daratumumab monotherapy for patients with relapsed or refractory natural killer/T-cell lymphoma, nasal type: an open-label, single-arm, multicenter, phase 2 study

**DOI:** 10.1186/s13045-020-01020-y

**Published:** 2021-02-15

**Authors:** Huiqiang Huang, Jun Zhu, Ming Yao, Tae Min Kim, Dok Hyun Yoon, Seok-Goo Cho, Hyeon Seok Eom, Soon Thye Lim, Su-peng Yeh, Yuqin Song, Yok Lam Kwong, Jin Seok Kim, Jie Jin, Yuankai Shi, HyeJin Kim, Min Qing, Tianyuan Zhou, Grace Gao, Zongqi Dong, Ming Qi, Won Seog Kim

**Affiliations:** 1 Sun Yat-Sen University Cancer Center, Guangzhou, China; 2 Beijing Cancer Hospital, Beijing, China; 3 National Taiwan University Hospital, Taipei, Taiwan; 4 Seoul National University Hospital, Seoul, South Korea; 5 Asan Medical Center, University of Ulsan College of Medicine, Seoul, South Korea; 6 Seoul St. Mary’s Hospital, Seoul, South Korea; 7 National Cancer Center, Goyang-si, South Korea; 8 National Cancer Centre Singapore, Singapore, Singapore; 9 China Medical University Hospital, Taichung, Taiwan; 10 Queen Mary Hospital, Pok Fu Lam, Hong Kong; 11 Yonsei University College of Medicine, Severance Hospital, Seoul, South Korea; 12 The First Affiliated Hospital of Zhejiang University Medical College, Hangzhou, China; 13 National Cancer Center/National Clinical Research Center for Cancer/Cancer Hospital, Chinese Academy of Medical Sciences & Peking Union Medical College, Beijing, China; 14 Janssen (China) Research & Development, Beijing, China; 15 Janssen (China) Research & Development, AP Center of Excellence, Translational Science, Shanghai, China; 16 Janssen Research & Development, LLC, Spring House, PA USA; 17 Division of Hematology/Oncology, Department of Medicine, Sungkyunkwan University School of Medicine, Samsung Medical Center, 81 Irwon-ro, Irwon-dong, Gangnam-gu, Seoul, 06351 South Korea

**Keywords:** Daratumumab, NK/T-cell lymphoma, CD38

## Abstract

**Background:**

Natural killer/T-cell lymphoma (NKTCL) is a disease with limited treatment options and poor outcomes. Daratumumab monotherapy demonstrated clinical activity in a single-patient case report. We present data from the primary analysis of a phase 2 study of daratumumab monotherapy in relapsed or refractory (R/R) NKTCL.

**Methods:**

This phase 2 study with Simon’s two-stage design evaluated daratumumab in patients with histologically confirmed extranodal NKTCL, nasal type, per WHO classification that was refractory to or relapsed after ≥ 1 line of chemotherapy, who were not candidates for other treatment modalities. All patients received daratumumab 16 mg/kg intravenously once weekly for Cycles 1 and 2, every other week for Cycles 3 through 6, and every 4 weeks thereafter until progression or unacceptable toxicity; all cycles were 28 days. The primary end point was objective response rate (ORR) based on blinded independent central review per Revised Criteria for Response Assessment of Hodgkin and non-Hodgkin Lymphoma (Lugano classification).

**Results:**

In total, 32 Asian patients received daratumumab. The ORR was 25.0% (95% confidence interval [CI] 11.5–43.4); all 8 responders had a partial response; and the median duration of response was 55.0 days (95% CI 29–339). At 10.2 months of median follow-up, median progression-free survival (PFS) was 53.0 days (95% CI 43–106); the 4-month PFS rate was 13.0%. Median overall survival (OS) was 141.0 days (95% CI 94–438); the 6-month OS rate was 42.9%. Nineteen (59.4%) patients had grade 3/4 treatment-emergent adverse events (TEAEs); the most common was thrombocytopenia (25.0%; *n* = 8). TEAEs leading to death occurred in 4 patients (death, respiratory failure, septic shock, and pneumonia); all were unrelated to daratumumab.

**Conclusions:**

In patients with R/R NKTCL, daratumumab monotherapy was well tolerated with no new safety concerns and achieved an ORR of 25.0%. However, no patients achieved complete response, and duration of response was short.

*Trial registration* ClinicalTrials.gov, NCT02927925. Registered 7 October 2016.

## Background

Natural killer/T-cell lymphomas (NKTCLs) are rare, Epstein–Barr virus (EBV)-associated distinct subtypes of peripheral T-cell lymphoma that are predominantly extranodal and of the nasal type [1]. Extranodal NKTCLs are aggressive and often lead to destructive facial lesions. The disease is more common in patients from Asian and Central/South American countries compared with North American and European countries (10% versus < 1% of non-Hodgkin’s lymphomas), partially because of regional differences in EBV exposure and pathogenesis [2]. Current therapeutic options for patients with relapsed or refractory (R/R) NKTCL include targeted agents such as immune checkpoint inhibitors (pembrolizumab or nivolumab), cytotoxic chemotherapies, or asparaginase-based regimens when not used in prior lines of therapy [3]. While evidence for novel therapies appears encouraging, these data are based on small retrospective or case studies [4, 5]. Currently, outcomes for patients with NKTCL are poor, with a 5-year overall survival (OS) rate for all patients with NKTCL of 32% and a median OS of 8 months [6, 7]. For patients with late-stage disease, outcomes are even worse; patients who relapse after asparaginase-containing regimens have a median OS of just a few months [8]. These data highlight that new treatment options are urgently needed for patients with advanced-stage NKTCL.

Daratumumab is a human IgGκ monoclonal antibody targeting CD38 with a direct on-tumor [9–12] and immunomodulatory [13–15] mechanism of action. Daratumumab is approved as monotherapy or combination therapy across lines of therapy for patients with multiple myeloma (MM) [16, 17]. Initial studies show that CD38 may be a novel therapeutic target for NKTCL therapy. In a study of 94 patients with NKTCL, 95% of tumor samples expressed CD38 and clinical data suggested that CD38 may be a novel prognostic biomarker [18]. Additionally, a case report described a heavily pretreated Asian woman with R/R NKTCL who achieved remission after receiving daratumumab [19].

We describe results from the phase 2 study, NKT2001 (ClinicalTrials.gov Identifier: NCT02927925), designed to evaluate daratumumab monotherapy in Asian patients with R/R extranodal NKTCL, nasal type. The interim analysis of the study was previously reported; daratumumab demonstrated high tolerability, and the pre-specified futility criterion (defined as ≤ 1 of 15 patients achieving complete response [CR]/partial response [PR] per protocol) was not met, warranting enrollment into stage 2 of the study [20]. Here, we present results from the primary analysis of this study.

## Methods

### Study design

This open-label, single-arm, multicenter, phase 2 study with Simon’s two-stage design enrolled patients with R/R extranodal NKTCL, nasal type, from 14 sites across South Korea, mainland China, Taiwan China, Hong Kong Special Administrative Region, and Singapore. A pre-specified interim analysis, which evaluated the safety and efficacy data from stage 1 of the study, occurred after 16 patients had received ≥ 1 dose of daratumumab and had ≥ 1 post-baseline disease evaluation; results were previously reported [20]. The primary analysis was performed after 32 patients were enrolled and at approximately 6 months after the last patient received the first dose of daratumumab.

The study was conducted in accordance with the principles of the Declaration of Helsinki, International Conference on Harmonisation Good Clinical Practice guidelines, and applicable regulatory requirements. Independent ethics committees or institutional review boards of all participating sites approved the study protocol and amendments. All patients provided written informed consent.

### Patients

Eligible patients were ≥ 18 years of age with histologically confirmed extranodal NKTCL, nasal type, per World Health Organization classification [21], that was refractory to or relapsed after achieving complete or partial remission on ≥ 1 line of chemotherapy, and were not candidates for other treatment modalities based on investigator assessment. Patients had ≥ 1 measurable site of disease that was positive for the uptake of ^18^F fluorodeoxyglucose (^18^F-FDG) in nodal or extranodal sites on positron emission tomography (PET) scan, an Eastern Cooperative Oncology Group performance status (ECOG PS) score of 0 to 2, and a life expectancy of ≥ 3 months. Patients were required to provide a fresh or archived formalin-fixed, paraffin-embedded tumor sample for biomarker evaluation. See supplementary methods in Additional file 1 for complete eligibility criteria.

### Procedures

The study consisted of a screening phase, a treatment phase, and a follow-up phase. The screening phase occurred within 21 days before Cycle 1 Day 1 (all cycles were 28 days) to verify eligibility for enrollment. During the treatment phase, patients received daratumumab 16 mg/kg by intravenous infusion once weekly during Cycles 1 and 2 (Days 1, 8, 15, and 22), then every other week during Cycles 3 through 6 (Days 1 and 15), and every 4 weeks thereafter until disease progression, unacceptable toxicity, or patient withdrawal. An end-of-treatment visit occurred within 30 days after the last dose of all study treatments. The follow-up phase occurred from treatment discontinuation to death, lost to follow-up, withdrawal of consent, or end of study, whichever occurred first. The end of study was defined as approximately 9 months after the last patient received the first dose.

Disease evaluations by radiological (computed tomography [CT; of the neck, chest, abdomen, and pelvis and any other location where disease was present at screening] or magnetic resonance imaging [to evaluate sites of disease that were not adequately imaged using CT]) and PET-CT (whole-body ^18^F-FDG PET-CT; skull base to the upper one-third of the thighs) scans occurred at screening, every 8 weeks (± 7 days) for the first 6 months, and every 16 weeks (± 7 days) thereafter until disease progression, withdrawal, or end of study. All imaging was reviewed by blinded independent central review (BICR) per pre-defined independent central review charter. The central reviewers assessed disease status based on the Revised Criteria for Response Assessment of Hodgkin and non-Hodgkin Lymphoma: Lugano classification [22]. Primary efficacy analysis was based on central review.

For measurement of serum concentrations of daratumumab, venous blood samples of approximately 5 mL were collected pre- and post-infusion. The generation of anti-daratumumab antibodies was assessed from blood samples collected pre-infusion (see supplementary methods in Additional file 1 for collection schedule). CD38 expression was assessed by immunohistochemistry methodology using rabbit anti-human CD38 monoclonal antibody (SP149; Cell Marque, Rocklin, CA, USA) as described previously [23]. Stains were reviewed and scored by a central panel of pathologists. Fresh tumor samples from core needle biopsy within 21 days of Cycle 1 Day 1 were preferred; if fresh samples were not available, archived formalin-fixed, paraffin-embedded blocks/slides were acceptable. See supplementary methods in Additional file 1 for additional biomarker assessment methods. Pharmacokinetic (PK), immunogenicity, and biomarker assessments were conducted at a central laboratory. Treatment-emergent adverse events (TEAEs) were recorded throughout the study and graded according to the National Cancer Institute Common Terminology Criteria for Adverse Events version 4.03 [24].

The primary analysis population for efficacy and safety was the safety population, which included all daratumumab-treated patients. The PK-evaluable population included all daratumumab-treated patients who had ≥ 1 post-infusion PK sample. The immunogenicity-evaluable population included all daratumumab-treated patients who had appropriate samples for detection of antibodies to daratumumab. The biomarker- and pharmacodynamic-evaluable population included all daratumumab-treated patients who had appropriate samples for valid assays.

### Outcomes

The primary end point was objective response rate (ORR; proportion of patients who achieved CR or PR) based on BICR per Revised Criteria for Response Assessment of Hodgkin and non-Hodgkin Lymphoma (Lugano classification) [22]. Secondary end points were CR rate, duration of response, time to response, progression-free survival (PFS), and overall survival (OS). See supplementary methods in Additional file 1 for definition of end points. Circulating EBV-DNA quantification was evaluated based on baseline and post-treatment plasma EBV-DNA levels. Safety assessments were evaluated based on TEAEs, clinical laboratory tests, vital sign measurements, physical examinations, electrocardiograms, and ECOG PS score. PKs were evaluated to determine the maximum serum daratumumab concentrations (C_max_), sampling time to reach maximum concentrations (t_max_), and area under the concentration–time curve (AUC). Biomarker evaluations included baseline tumor tissue CD38 expression, and baseline and post-treatment peripheral blood B-cell and natural killer (NK)–cell counts.

### Statistical analysis

This study evaluated the effect of daratumumab on ORR using Simon’s two-stage design. The null hypothesis was that ORR was at most 15%, and the alternative hypothesis was that ORR was at least 30%. With a one-sided alpha of 10% and a power of 78%, a total of 32 patients (both stages) were required to enroll in the study. In stage 1, a protocol-specified futility analysis occurred after approximately 15 patients received ≥ 1 dose of daratumumab and had ≥ 1 post-baseline disease evaluation. The futility criterion for ORR was defined as, at most, 1 of 15 patients with CR/PR. Because the futility criterion was not met [20], stage 2 continued to enroll patients and was not terminated. Per protocol, if the study proceeded to stage 2 with a total of 32 patients, the null hypothesis would be rejected if ≥ 8 responses were observed. Additional enrollment of patients into an expansion phase was planned if data from stage 1 and 2 were positive. See supplementary methods in Additional file 1 for additional statistical analyses methods.

## Results

### Patients

A total of 32 patients were enrolled and treated with daratumumab. Patient demographics and baseline disease characteristics are summarized in Table 1. The median (range) patient age was 56.0 (22–78) years with 24 (75.0%) patients younger than 65 years of age. Twenty-three (71.9%) patients were male, and 29 (90.6%) patients had an ECOG PS score of 0 or 1. All 32 enrolled patients were Asian. Median time from initial NKTCL diagnosis was 24.0 months (range 3.1–185.4), and median plasma EBV-DNA at baseline was 3800.0 (range 0–11,291,151) kIU/L (*n* = 31). The majority of patients were high or intermediate risk based on the prognostic index of NK lymphoma (PINK; 23/31 [74.2%]) and PINK–Epstein–Barr virus (PINK-E; 21/30 [70.0%]). Of the 22 patients with evaluable CD38 expression data by immunochemistry, 11 patients had baseline CD38 expression values ≥ 50% and the remaining 11 had CD38 expression values ≥ 0% to 49%.

**Table 1:** Patient demographics and baseline disease characteristics

Characteristic	Daratumumab 16 mg/kg (*n* = 32)
Age	*n* = 32
Median (range), years	56.0 (22–78)
< 65 years, *n* (%)	24 (75.0)
65–74 years, *n* (%)	7 (21.9)
≥ 75, *n* (%)	1 (3.1)
Sex, *n* (%)	*n* = 32
Male	23 (71.9)
Female	9 (28.1)
Asian, *n* (%)	32 (100)
ECOG PS score, *n* (%)	*n* = 32
0	14 (43.8)
1	15 (46.9)
2	3 (9.4)
Median (range) time since diagnosis, months	24.0 (3.1–185.4)
Percentage of CD38 expression	*n* = 22
Median (range), %	45.0 (0–100)
≥ 0% to 49%, *n* (%)	11 (50.0)
≥ 50%, *n* (%)	11 (50.0)
H-score of CD38 expression	*n* = 22
Median (range)	67.5 (0–300)
≥ 0 to 49, *n* (%)	10 (45.5)
≥ 50, *n* (%)	12 (54.5)
Plasma EBV-DNA, kIU/L	*n* = 31
Median (range)	3800.0 (0–11,291,151)
β_2_ microglobulin, mg/L	*n* = 31
Median (range)	3.0 (1.7–11.5)
Site of disease involvement at initial diagnosis, *n* (%)	*n* = 31
Upper aerodigestive tract only	17 (54.8)
Extra-upper aerodigestive tract only	5 (16.1)
Both	9 (29.0)
PINK, *n* (%)	*n* = 31
Low	8 (25.8)
Intermediate	15 (48.4)
High	8 (25.8)
PINK-E, *n* (%)	*n* = 30
Low	9 (30.0)
Intermediate	13 (43.3)
High	8 (26.7)
Median (range) prior lines of therapy	2 (1–8)
Prior lines of therapy, *n* (%)	*n* = 32
1	11 (34.4)
2	8 (25.0)
≥ 3	13 (40.6)
Prior therapies, *n* (%)	*n* = 32
L-asparaginase–containing regimen	27 (84.4)
Radiotherapy	21 (65.6)
Cancer-related surgery/procedure	15 (46.9)
Anthracycline-based regimen	8 (25.0)
Autologous stem cell transplant	4 (12.5)

EBV, Epstein–Barr virus; ECOG PS, Eastern Cooperative Oncology Group performance status; PINK, prognostic index of natural killer lymphoma; PINK-E, prognostic index for natural killer cell lymphoma–Epstein–Barr virus

At the clinical cutoff date (9 October 2019), the median follow-up was 10.2 months and 30 (93.8%) patients had discontinued treatment due to progressive disease (22 patients [68.8%]), withdrawal of consent (4 [12.5%]), physician decision (3 [9.4%] patients), and adverse events (1 [3.1%]). The remaining 2 (6.3%) patients continued receiving treatment per protocol. The median duration of daratumumab treatment was 38.5 days (range 1–408), the median number of daratumumab infusions was 6.0, and the median number of treatment cycles was 2 (range 1–14).

### Efficacy

**Fig. 1:**
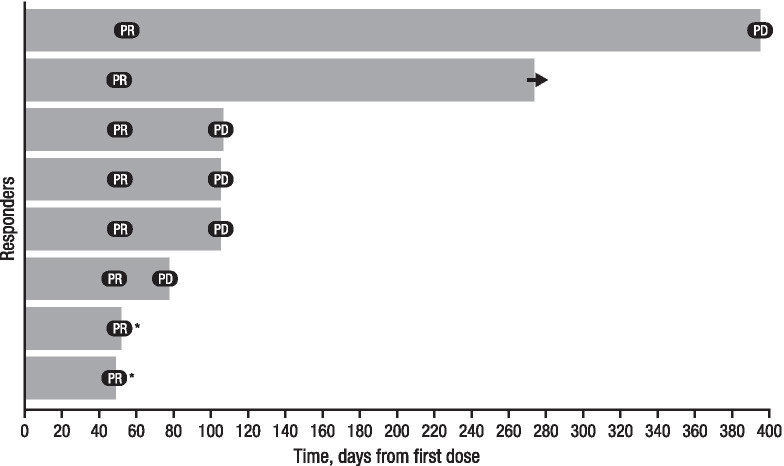
Swim lane plot for duration of response based on central review in daratumumab-treated responders. Responses are shown (PR). Five patients had progressive disease (PD) and 2 patients discontinued treatment based on investigator’s assessment of PD (*) and no further assessment of response occurred due to withdrawal from study. The arrow indicates ongoing treatment at clinical cutoff date (1 patient). PD, progressive disease; PR, partial response

The primary end point, ORR based on BICR per Revised Criteria for Response Assessment of Hodgkin and non-Hodgkin Lymphoma (Lugano classification) [22], was 25.0% (95% CI 11.5–43.4; Table 2). Among the 32 treated patients, no patient achieved CR, and all 8 (25.0%) patients with a response achieved PR. The clinical benefit rate (CR + PR + stable disease) was 43.8% (95% CI 26.4–62.3). The median (range) time to first response was 52 (49–57) days, and the median duration of response was 55 days (95% CI 29–339). A swim lane plot for duration of response is shown in Fig. 1. At clinical cutoff, 5 (62.5%) of the 8 responders had disease progression, 2 (25.0%) discontinued treatment without progression, and 1 (12.5%) patient continued treatment. ORR by investigator assessment is reported in the supplementary results (see Additional file 1).

**Table 2:** Overall best response among all treated patients by BICR

Response	Daratumumab 16 mg/kg (*n* = 32)		
	No.	%	95% CI
ORR	8	25.0	11.5–43.4
Clinical benefit rate (CR + PR + stable disease)	14	43.8	26.4–62.3
CR	0	0	
PR	8	25.0	
Stable disease	6	18.8	
PD	14	43.8	
NE	4	12.5	

BICR, blinded independent central review; CI, confidence interval; CR, complete response; NE, not evaluable; ORR, objective response rate; PD, progressive disease; PR, partial response

**Fig. 2:**
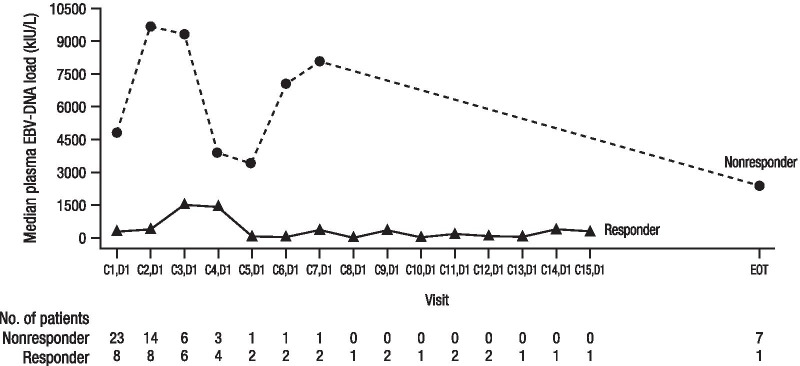
Summary of plasma EBV-DNA levels over time. Median plasma EBV-DNA loads (kIU/L) over time are shown for responders and nonresponders in the pharmacodynamic analysis population. Responses were based on central review. Nonevaluable patients were considered nonresponders. C, Cycle; D, Day; EBV, Epstein–Barr virus; EOT, end of treatment

Median plasma EBV-DNA levels over time are shown in Fig. 2. Among the 25 patients evaluable for plasma EBV-DNA change from baseline, 4 (50.0%) of 8 responders had an EBV-DNA load reduction from baseline of ≥ 50% at any post-baseline visit, and 3 (13.6%) of 17 evaluable nonresponders had a load reduction ≥ 50% at any post-baseline visit.

**Fig. 3:**
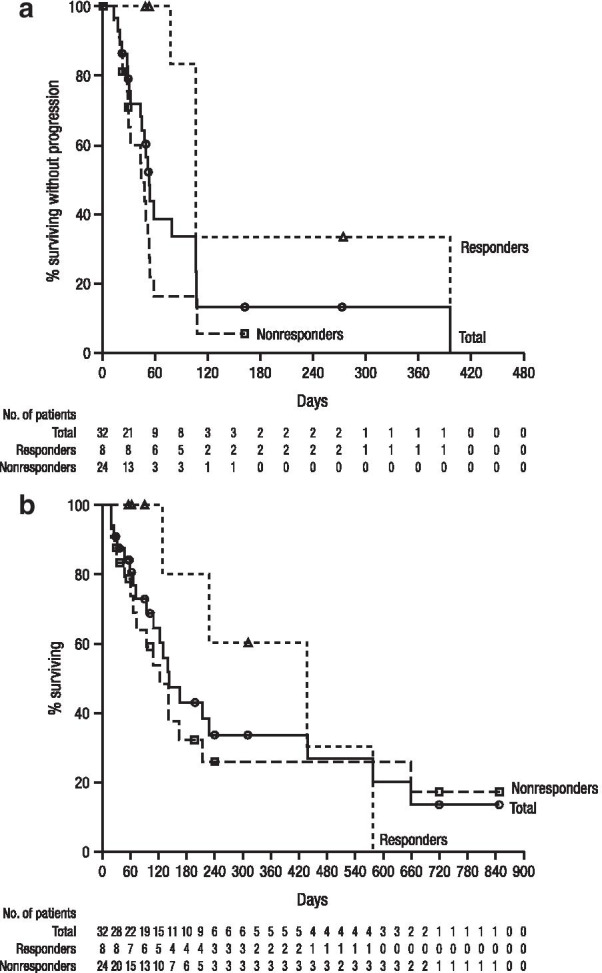
**a** Progression-free survival and **b** overall survival in patients with relapsed or refractory natural killer/T-cell lymphoma treated with daratumumab. Kaplan–Meier curves are shown for the overall population (all-treated patients; *n* = 32) and for responders (*n* = 8) and nonresponders (*n* = 24). Responses were based on central review. Nonevaluable patients were considered nonresponders

At clinical cutoff, a total of 23 (71.9%) of patients had progressive disease or died; the median PFS was 53 days (95% CI 43–106), with a 4-month PFS rate of 13.0% (95% CI 3.3–29.5; Fig. 3a). A total of 20 (62.5%) deaths were observed; the median OS was 141 days (95% CI 94–438), with a 6-month OS rate of 42.9% (95% CI 23.5–61.0; Fig. 3b).

### Safety

All patients had ≥ 1 TEAE. The most common any-grade TEAEs were pyrexia (21 [65.6%] patients); thrombocytopenia, anemia, and increased alanine aminotransferase (9 [28.1%] each); and neutropenia, headache, increased aspartate aminotransferase, and chills (8 [25.0%] each; Table 3). A total of 19 (59.4%) patients had grade 3/4 TEAEs, most commonly thrombocytopenia (8 [25.0%] patients), neutropenia (6 [18.8%]), and anemia and leukopenia (5 [15.6%] each; Table 3). Serious AEs (SAEs) were reported in 17 (53.1%) patients, with approximately half (8 [25.0%]) considered related to daratumumab. Pyrexia (5 [15.6%]) was the only SAE reported in ≥ 10% of patients. One patient reported a TEAE leading to treatment discontinuation (death; not related to daratumumab). TEAEs leading to death occurred in 4 patients (death, respiratory failure, septic shock, and pneumonia; one of these patients died 30 days after the last daratumumab dose, while on subsequent therapy). All TEAEs leading to death were considered unrelated to daratumumab.

**Table 3:** Most common any-grade (≥ 20% of patients) and grade 3/4 (≥ 10% of patients) TEAEs

Event, *n* (%)	Daratumumab 16 mg/kg (*n* = 32)	
	Any grade	Grade 3/4
Any TEAE	32 (100.0)	19 (59.4)
Pyrexia	21 (65.6)	4 (12.5)
Thrombocytopenia	9 (28.1)	8 (25.0)
Anemia	9 (28.1)	5 (15.6)
Increased alanine aminotransferase	9 (28.1)	1 (3.1)
Neutropenia	8 (25.0)	6 (18.8)
Headache	8 (25.0)	1 (3.1)
Increased aspartate aminotransferase	8 (25.0)	1 (3.1)
Chills	8 (25.0)	0
Leukopenia	7 (21.9)	5 (15.6)
Fatigue	7 (21.9)	1 (3.1)

TEAE, treatment-emergent adverse event

Infusion-related reactions (IRRs) were reported in 20 (62.5%) patients. All IRRs occurred during the first cycle and were generally mild (grades 1–2). Two (6.3%) patients had grade 3 IRRs (1 patient with urticaria and 1 patient with hypertension and hypotension); no grade 4 or 5 IRRs were reported***.*** No patients discontinued daratumumab because of IRRs.

### Pharmacokinetics and immunogenicity

PK data were available and reported in 31 patients. PK parameters following the first infusion were calculated in 19 patients with a relatively intensive PK profile. Following the first infusion, mean (standard deviation [SD]) C_max_ was 267 (56.3) μg/mL (*n* = 19) and mean (SD) AUC_0–7 days_ was 24,751 (5849) hours × μg/mL (*n* = 17). Mean (SD) pre-dose serum daratumumab concentration at Cycle 3 Day 1 (maximum trough concentration [C_trough_]) was 450 (179) μg/mL (*n* = 9). No obvious trend or difference in PK was observed between PK-evaluable responders and nonresponders following the first infusion, and the pre-dose Cycle 3 Day 1 serum concentrations (maximum C_trough_) largely overlapped for responders and nonresponders. Of the 26 immunogenicity-evaluable patients, none were positive for anti-daratumumab antibodies.

### Biomarkers

Baseline tumor CD38 expression levels were evaluated by immunochemistry in 22 patients (responders, *n* = 6; nonresponders, *n* = 16). There was no clear trend between baseline CD38 expression and response to daratumumab. The mean (SD) and median (range) percentages of tumor cells that expressed CD38 at baseline were 56.7% (39.3) and 60.0% (0–100), respectively, in responders and 41.6% (31.9) and 40.0% (0–100) in nonresponders (Additional file 2: Fig. S1). The mean (SD) and median (range) H-scores of tumor cells that expressed CD38 were 95.0 (76.9) and 80.0 (0–190), respectively, in responders and 80.3 (84.8) and 60.0 (0–300) in nonresponders.

Major immune cell types in peripheral blood of patients were measured by flow cytometry. Baseline B-cell (CD45^bright^SSC^low^CD3^−^CD19^+^) counts were measured in 29 patients (responders, *n* = 8; nonresponders, *n* = 21). B-cell counts at baseline were higher in responders than in nonresponders; the mean (SD) baseline B-cell counts were 233.1 (172.5) × 10^6^/L in responders and 54.4 (42.0) × 10^6^/L in nonresponders (Additional file 2: Fig. S2). Overall, there were no substantial changes in B-cell counts among responders at Cycle 2 Day 1 after daratumumab treatment, and by the end of treatment, mean (SD) B-cell counts were 102.0 (92.32) × 10^6^/L in responders (*n* = 4) and 32.8 (28.78) × 10^6^/L in nonresponders (*n* = 11).

Compared with other immune cell populations in peripheral blood, NK cells express high levels of CD38 [13]. Anti-CD38 treatment led to consistent and acute reductions of NK cells in the peripheral blood of MM patients [25]. To confirm whether daratumumab showed similar pharmacodynamics in NKTCL patients, baseline total absolute NK-cell (CD45^+^CD3^−^CD16^+^CD56^+^) counts were measured in 29 patients (responders, *n* = 8; nonresponders, *n* = 21). The baseline total NK-cell counts were similar between responders and nonresponders. Regardless of response, a decrease in NK-cell percentage and NK-cell counts was observed after one cycle of daratumumab (*n* = 19). The mean (SD) change in NK-cell percentage from baseline to Cycle 2 Day 1 (*n* = 19) was − 15.3% (9.7), and the mean (SD) change in NK-cell counts from baseline to Cycle 2 Day 1 was –0.18 (0.16) × 10^9^/L (Additional file 2: Fig. S3). The mean (SD) change in NK-cell percentage and counts from baseline was similarly reduced at the end of treatment (–16.3% [9.0] and –0.20 [0.14], respectively; *n* = 15).

## Discussion

This phase 2 study evaluated daratumumab 16 mg/kg monotherapy in Asian patients with R/R NKTCL, nasal type. In stage 1 of this study, the interim analysis of 16 patients who received ≥ 1 daratumumab dose demonstrated an ORR of 35.7% among the 14 response-evaluable patients, which did not meet the pre-specified futility criteria, and therefore, the study extended into stage 2 [20]. In stage 2 of the study, we report that a total of 32 patients enrolled and daratumumab monotherapy demonstrated an ORR of 25.0% based on BICR assessment, with a clinical benefit rate of 43.8%. No patients achieved CR, and the duration of response was short (55 days). With 10.2 months of median follow-up, the median PFS was 53 days and the median OS was 141 days. These data suggest that daratumumab monotherapy has modest activity as a single agent against NKTCL but may not be sufficient to treat patients with aggressive features, especially those who have poor prognosis features. Notably, on the basis of the prognostic index of NK lymphoma (PINK) and PINK–E, the majority of patients in this study were intermediate and high risk.

No randomized clinical trials have been conducted to compare treatment regimens in patients with R/R NKTCL. In a single-arm phase 2 study of the AspaMetDex regimen (L-asparaginase/methotrexate/dexamethasone) in patients with R/R extranodal NKTCL and no prior asparaginase therapy, the ORR after three cycles of treatment was 77.8% (*N* = 18) [26]. In the subset of patients with R/R extranodal NKTCL from a single-arm phase 2 study of the SMILE regimen (dexamethasone/methotrexate/ifosfamide/L-asparaginase/etoposide), the ORR after two cycles of treatment was 77.8% (*N* = 18) [27]. Another asparaginase-based combination therapy, MEDA (methotrexate/etoposide/dexamethasone/L-asparaginase), demonstrated a similar ORR (76.9%) in a retrospective analysis (*N* = 13) [28]. Undoubtedly, these response rates are higher than those seen with daratumumab for R/R NKTCL (ORR, 25.0%); however, the majority of patients (84%) in the present study received a prior L-asparaginase–containing regimen, so lower response rates would be expected. In a retrospective study of patients (*N* = 20) with extranodal NKTCL who had progression on an L-asparaginase–containing regimen, gemcitabine with or without chemotherapy led to an ORR of 40%, with 8 patients achieving CR [8]. More recently, promising antitumor activity was seen with programmed cell death protein 1 (PD-1) inhibitors among patients with NKTCL who failed asparaginase-based combination therapy. In a retrospective study of 7 patients, pembrolizumab monotherapy was associated with high response rates (2 patients achieved CR in all parameters, 3 achieved clinical and radiologic CR, and 2 achieved molecular remission) [4]. Similarly, a case study of nivolumab monotherapy among 3 patients with NKTCL who failed on L-asparaginase–based therapy demonstrated encouraging results among patients with very poor prognoses [5]. Additional rigorous studies should be conducted for PD-1 inhibitors and other therapeutic agents for the treatment of R/R NKTCL.

In R/R NKTCL patients, daratumumab monotherapy was well tolerated. The TEAEs reported in this study were consistent with the known safety profile observed in patients with MM [29–31]. The most common grade 3/4 TEAEs were predominantly cytopenias. There was no death or treatment discontinuation due to TEAEs related to daratumumab. IRRs occurred in 62.5% of patients, all in the first cycle of therapy. IRRs were generally mild; 2 patients had grade 3 IRRs, and none were grade 4 or 5.

The PK profile of daratumumab in R/R NKTCL patients was consistent with previous studies of daratumumab in patients with MM [16]. Following daratumumab administration, accumulation of daratumumab continued throughout the weekly dosing. In patients with MM, the maximum daratumumab C_trough_ (Cycle 3 Day 1 pre-dose; the end of weekly monotherapy dosing) was previously shown to have the strongest correlation with ORR based on population PK and exposure–response analyses [32]. Thus, in this study, maximum C_trough_ concentrations were compared between responders and nonresponders; no obvious trend was observed. Additionally, C_max_ and AUC following the first infusion were generally comparable between responders and nonresponders. No patients were positive for anti-daratumumab antibodies, which indicates a low risk of daratumumab immunogenicity and is consistent with prior reports [31].

In MM, daratumumab has been associated with a significant treatment-related reduction of NK cells that does not impact drug efficacy or safety [15, 25]. This study showed that daratumumab was associated with a reduction in NK cells, consistent with the known sensitivity of this cell population to daratumumab [13]; however, baseline total NK-cell counts and percentage of NK cell decrease in peripheral blood were not associated with response to daratumumab for NKTCL. Immune profiling studies confirmed the presence of persisting NK cells in the NKTCL patients after daratumumab treatment (data not shown), consistent with results reported for MM [13–15]. Baseline CD38 expression level on NKTCL tumor tissue also had no direct association with response, suggesting that a complex combination of NK-cytotoxic activity and tumor microenvironment modulation functions of daratumumab needs to be considered to dissect the contribution of antitumor effects. Interestingly, baseline B-cell counts in peripheral blood were higher in responders versus nonresponders, and nonresponders trended toward a decrease in B-cell counts after daratumumab treatment, yet further analysis would be required to explore whether baseline B-cell count is a predictive biomarker for daratumumab in NKTCL. The small sample size and inconsistent number of patients with evaluable samples at different time points limit the interpretation of PK and biomarker results.

## Conclusions

In conclusion, single-agent daratumumab 16 mg/kg monotherapy in patients with R/R NKTCL achieved an ORR of 25.0% and duration of response was short, and the safety and PK analyses were consistent with previously published data of daratumumab in MM. Although these results demonstrated limited clinical benefit of daratumumab monotherapy in NKTCL, exploration of daratumumab in combination with novel therapy for the treatment of NKTCL may be an area of interest in subsequent research. Whether daratumumab can be combined with agents used for the treatment of NKTCL remains to be seen.

## Supplementary Information

**Additional file 1:** Supplementary methods and results.

**Additional file 2:** Supplementary figures.

## Data Availability

The data sharing policy of Janssen Pharmaceutical Companies of Johnson & Johnson is available at https://www.janssen.com/clinical-trials/transparency. As noted on this site, requests for access to the study data can be submitted through Yale Open Data Access (YODA) Project site at http://yoda.yale.edu.

## Abbreviations

^18^F-FDG: ^18^F fluorodeoxyglucose
AUC: Area under the concentration–time curve
BICR: Blinded independent central review
C: Cycle
CI: Confidence interval
C_max_: Maximum concentration
CR: Complete response
CT: Computed tomography
C_trough_: Maximum trough concentration
D: Day
EBV: Epstein–Barr virus
ECOG PS: Eastern Cooperative Oncology Group performance status
EOT: End of treatment
IRR: Infusion-related reaction
MEDA: Methotrexate/etoposide/dexamethasone/L-asparaginase
MM: Multiple myeloma
NE: Not evaluable
NK: Natural killer
NKTCL: Natural killer/T-cell lymphoma
ORR: Objective response rate
OS: Overall survival
PD: Progressive disease
PD-1: Programmed cell death protein 1
PET: Positron emission tomography
PFS: Progression-free survival
PINK: Prognostic index of natural killer lymphoma
PINK-E: PINK–Epstein–Barr virus
PK: Pharmacokinetic
PR: Partial response
R/R: Relapsed or refractory
SAE: Serious adverse event
SD: Standard deviation
TEAE: Treatment-emergent adverse event
*t*_max_: Time to reach maximum concentration
