# Supplementary material for: Daratumumab monotherapy for patients with relapsed or refractory natural killer/T-cell lymphoma, nasal type: an open-label, single-arm, multicenter, phase 2 study
Source: J Hematol Oncol. 2021 Feb 15;14:25. doi: 10.1186/s13045-020-01020-y (PMC7885403; doi:10.1186/s13045-020-01020-y)
Supplement: Supplementary file 1 — Additional file 1: Supplementary methods and results. [file 13045_2020_1020_MOESM1_ESM.docx]

# Additional file 1: Supplementary methods and results

## Supplementary methods

### Inclusion criteria

Eligible patients were ≥18 years of age with histologically confirmed extranodal natural killer/T-cell lymphoma (NKTCL), nasal type, per World Health Organization classification [1], that was refractory to or relapsed after achieving complete or partial remission on ≥1 line of chemotherapy, and were not candidates for other treatment modalities. Patients had ≥1 measurable site of disease that was positive for the uptake of ^18^F fluorodeoxyglucose in nodal or extranodal sites on positron emission tomography (PET) scan, had Eastern Cooperative Oncology Group performance status score of 0 to 2, and had a life expectancy ≥3 months. Patients were required to have the following clinical laboratory values: hemoglobin ≥7.5 g/dL without transfusion support within 7 days, absolute neutrophil count ≥0.75 × 10^9^/L without growth factor support within 7 days, platelet count ≥50 × 10^9^/L without transfusion support within 7 days, aspartate and alanine aminotransferase levels ≤2.5 × the upper limit of normal (ULN), total bilirubin level ≤1.5 × ULN, and calculated creatinine clearance ≥30 mL/min (using the Cockcroft-Gault formula). Patients were required to provide a fresh or archived formalin-fixed, paraffin-embedded tumor sample for biomarker evaluation.

### *Exclusion criteria*

Exclusion criteria were clinical symptoms of central nervous system involvement, prior daratumumab or other anti-C38 therapies, chemotherapy or radiotherapy within 3 weeks before the first dose of study drug, corticosteroids as part of disease treatment within 2 weeks before the first dose of study drug, autologous stem cell transplant or allogenic stem cell transplant within 12 weeks before the first dose of study drug, history of active malignancy other than NKTCL within 2 years before the first dose, clinically significant cardiovascular disease, chronic obstructive pulmonary disease with a forced expiratory volume in 1 second <50% of predicted normal, uncontrolled asthma, moderate or severe persistent asthma within 2 years, seropositivity for human immunodeficiency virus, hepatitis B, or hepatitis C, unresolved or unstable serious toxicity from prior investigational drug or cancer treatment, concurrent medical condition that is likely to interfere with study or would be a hazard for participating patient, known allergies or hypersensitivity to corticosteroids, monoclonal antibodies, human proteins, or their excipients, known or suspected to not be able to comply with study protocol, invasive investigational medical device within 4 weeks before the screening period, vaccination with live attenuated vaccines within 4 weeks before first dose of study drug, and pregnancy or lactation.

### *Blood sample collection schedule*

For measurement of serum concentrations of daratumumab, venous blood samples of approximately 5 mL were collected pre- and post-infusion as follows: Cycle 1 (pre- and post-infusion on Day 1 and pre-infusion on Days 8 and 22); Cycle 2 (pre-infusion on Day 1 and pre- and post-infusion on Day 22); Cycle 3 (pre- and post-infusion on Day 1); Cycles 4 through 7, 9, 11 through 13, 15, and 17 onwards (pre-infusion on Day 1); samples were also taken at the end-of-treatment visit and at the 4- and 8-week follow-up visits. The generation of anti-daratumumab antibodies was assessed from blood samples collected pre-infusion on Day 1 of Cycles 1, 7, and 12, at the end-of-treatment visit, and at the 4- and 8-week follow-up visits.

### Biomarker assessment

Fresh tumor samples from core needle biopsy within 21 days of Cycle 1 Day 1 were preferred; if not available, archived formalin-fixed, paraffin-embedded blocks/slides were acceptable. The major lymphocyte subsets have been identified by antigenic determinants on the cell, i.e., T cells (CD3^+^), B cells (CD19^+^), NK cells (CD3^-^, CD16^+^/56^+^), helper/inducer T cells (CD3^+^, CD4^+^), and cytotoxic T cells (CD3^+^, CD8^+^). Cell counts were determined using BD Trucount^TM^ beads on BD FACSCanto^TM^ II flow cytometer (BD Biosciences, San Jose, CA, USA) from whole blood samples collected pre-infusion on Day 1 of Cycles 1, 3, and 7 and at the end-of-treatment visit. Circulating plasma EBV-DNA quantification was monitored monthly and at the end-of-treatment visit. Plasma EBV-DNA titer was measured by quantitative polymerase chain reaction with EBNA-1 and EBER targeting TaqMan^TM^ assays (Viracor-IBT Laboratories, Summit, MO, USA). The limit of detection of the assay in plasma was determined to be 12 copies/mL.

### *Definition of efficacy end points*

Objective response rate (ORR) was defined as the proportion of patients who achieved complete response (CR) or partial response (PR) per Revised Criteria for Response Assessment of Hodgkin and non-Hodgkin lymphoma [2]. CR rate was defined as the proportion of patients who achieved CR and were analyzed similarly as the primary end point, ORR. Duration of response was defined as duration from the date of the initial documentation of a response to the date of first documented evidence of progressive disease (or relapse for patients who experienced CR). For patients who had not progressed/relapsed, duration of response was to be censored at the last adequate tumor assessment. Progression-free survival (PFS) was defined as the duration from the date of the first daratumumab dose to the date of progression/relapse, or death, whichever came first. For patients who were still alive without progression/relapse, PFS was to be censored at the last adequate tumor assessment. Overall survival (OS) was defined as the duration from the date of the first daratumumab dose to the date of death. For patients who were still alive without progression/relapse, OS was to be censored at the last date known to be alive. Time to response was defined as the duration from the date of the first dose of daratumumab to the earliest date that a response (CR/PR) was first documented. For nonresponders, it was to be censored at the date of progression/relapse or the date of the last adequate disease assessment, whichever came first.

### Statistical analysis

ORRs were reported with two-sided 95% exact confidence intervals (CIs), and the number and percentage of patients in each response category were descriptively tabulated. For time-to-event end points Kaplan–Meier estimates were presented, along with medians and 95% CIs. Analyses for time to response and duration of response were for responders only. Descriptive statistics were used to summarize PK parameters, and mean serum daratumumab concentration time profiles were provided.

## Supplementary results

Based on investigator assessment, the ORR was 12.5% (95% CI 3.5-29.0), with 4 of 32 patients achieving PR or CR. The difference between the ORR reported by blinded independent central review (BICR) and investigator assessment was mainly due to discrepancies in PET-CT findings. The ORR by BICR was later confirmed by investigators after a quality control review of discordance cases.

## References

1. Swerdlow SH, Campo E, Pileri SA, Harris NL, Stein H, Siebert R, et al. The 2016 revision of the World Health Organization classification of lymphoid neoplasms. Blood. 2016;127:2375–90.
2. Cheson BD, Fisher RI, Barrington SF, Cavalli F, Schwartz LH, Zucca E, et al. Recommendations for initial evaluation, staging, and response assessment of Hodgkin and non-Hodgkin lymphoma: the LUGANO classification. J Clin Oncol. 2014;323059–68.
