# Supplementary material for: Daratumumab monotherapy for patients with relapsed or refractory natural killer/T-cell lymphoma, nasal type: an open-label, single-arm, multicenter, phase 2 study
Source: J Hematol Oncol. 2021 Feb 15;14:25. doi: 10.1186/s13045-020-01020-y (PMC7885403; doi:10.1186/s13045-020-01020-y)
Supplement: Supplementary file 2 — Additional file 2: Supplementary figures. [file 13045_2020_1020_MOESM2_ESM.pdf]

Additional file 2: Supplementary figures

Fig. S1 Baseline CD38 expression (% positive) by immunohistochemistry in tumor biopsy samples by response. The line in the middle of each shaded box represents the median. The diamond represents the mean. Each shaded box represents the values in the interquartile range. The outer lines represent the minimum and maximum. Responses were based on central review. Nonevaluable patients were considered nonresponders.

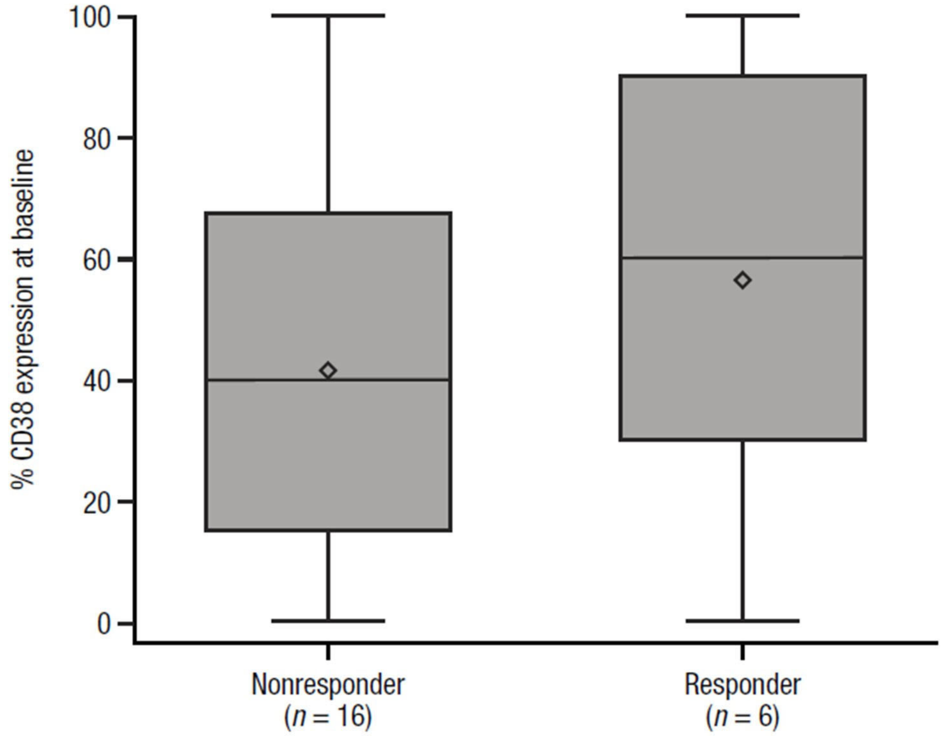

Fig. S2 Baseline B-cell counts in peripheral blood by response. The line in the middle of each shaded box represents the median. The diamond represents the mean. Each shaded box represents the values in the interquartile range. The outer lines represent the minimum and maximum. Responses were based on central review. Nonevaluable patients were considered nonresponders.

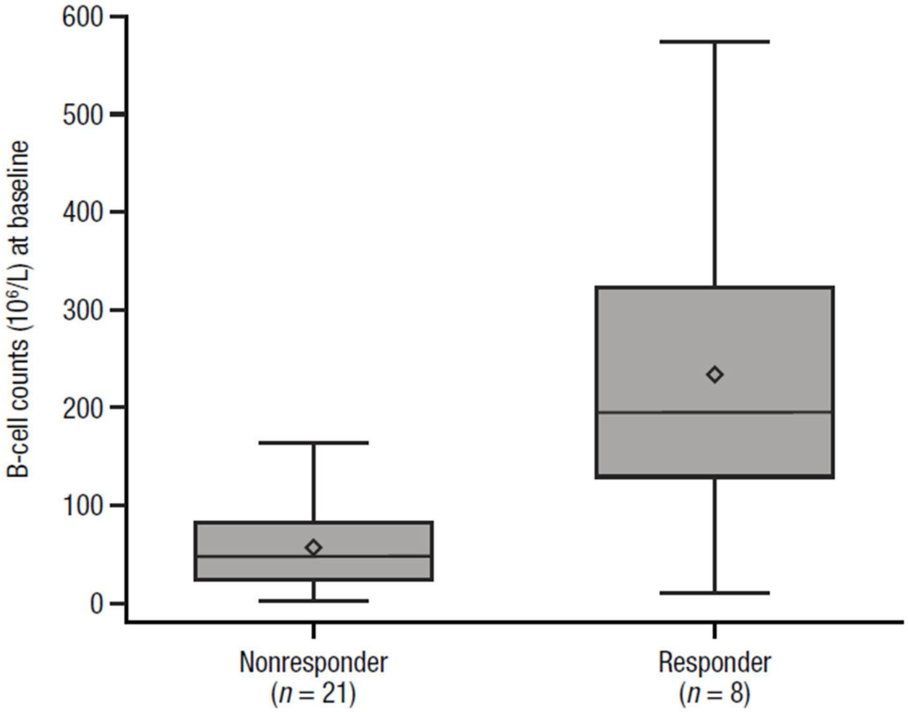

Fig. S3 Total natural killer-cell counts (CD45$^+$CD3$^-$CD16$^+$CD56$^+$) over time in peripheral blood by response. Responses were based on central review. Nonevaluable patients were considered nonresponders. C, Cycle; D, Day; EOT, end of treatment.

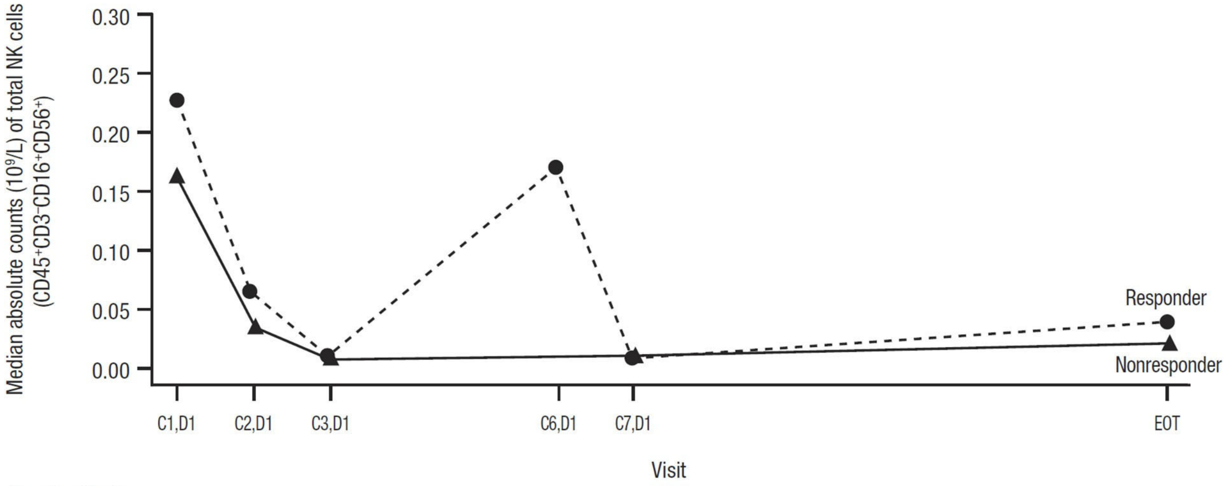

No. of patients						
Responder	8	7	2	1	1	4
Nonresponder	21	12	2	0	1	11
